# Higher Dietary Inflammatory Index Scores Increase the Risk of Diabetes Mellitus: A Meta-Analysis and Systematic Review

**DOI:** 10.3389/fendo.2021.693144

**Published:** 2021-08-11

**Authors:** Qing-Qing Tan, Xin-Yi Du, Chen-Lin Gao, Yong Xu

**Affiliations:** ^1^Department of Endocrinology and Metabolism, The Affiliated Hospital of Southwest Medical University, Luzhou, China; ^2^Cardiovascular and Metabolic Diseases Key Laboratory of Luzhou, The Affiliated Hospital of Southwest Medical University, Luzhou, China; ^3^Sichuan Clinical Research Center for Nephropathy, The Affiliated Hospital of Southwest Medical University, Luzhou, China

**Keywords:** body mass index, diabetes mellitus, diet, dietary inflammatory index, inflammation

## Abstract

The relationship between dietary inflammatory index (DII) scores and the risk of diabetes mellitus (DM) is unclear; therefore, a systematic review and meta-analysis of the current published literature was conducted. Relevant studies published online (PubMed, Embase, and Web of Science) until February 1, 2021 were identified for review. The initial search yielded 13 reports, and after perusing their titles, abstracts, and full texts, 5 studies were deemed appropriate for inclusion in the systematic review and meta-analysis. Individuals with higher DII scores (representing a more proinflammatory diet) had a higher risk of DM (pooled odds ratio 1.32, 95% confidence interval 1.01–1.72, I^2^ 58.6%, *p* < 0.05). Although the current meta-analysis indicated a trend toward a positive association between DII and DM, further evidence—especially from larger prospective studies in different countries—is needed to clarify this association.

## Introduction

Diabetes mellitus (DM) has become a global epidemic that affects millions of people worldwide. Dietary intervention is obviously effective in the treatment of DM and its associated complications. Multiple factors may contribute to the development of DM, and among them, chronic inflammation is widely considered an underlying pathophysiological mechanism involved in the development of DM (1). Studies suggest that multiple factors that affect inflammation may be associated with DM, such as sex, age, smoking, alcohol, physical activity, the application of some medications, and diet (2). The premise that diet can increase or reduce inflammation has been extensively investigated (3–5), but the degree to which modulating diet can have desirable effects in DM is unclear. It is therefore important to clarify relationships between diet, inflammation, and DM.

The dietary inflammatory index (DII) is a useful tool created by Cavicchia et al. (6) in 2009 and Shivappa et al. (7) in 2014 that has been developed for use in different populations. It can be used to estimate the overall inflammatory potential of an individual’s diet by utilizing a 24-h dietary record interview or food frequency questionnaires (FFQs) (7). The DII is based on 1943 eligible articles that reported the effects of 45 dietary parameters on serum levels of six biomarkers; the proinflammatory biomarkers interleukin (IL)-1β, IL-6, tumor necrosis factor alpha, and C-reactive protein, and the anti-inflammatory biomarkers IL-4 and IL-10 (7). Positive DII scores indicate a proinflammatory diet that significantly increases serum proinflammatory biomarker levels, whereas negative DII scores indicate the contrary. Therefore, DII scores can constitute a quantitative measure for the assessment of the relationships between diet and health outcomes. They can be used to identify the associations between diet and parameters ranging from serum concentrations of inflammatory factors to various types of chronic disease indicators (8) such as cardiovascular diseases (9), metabolic syndrome (10), obesity (11), cancer (12), and DM (13).

The relationship between the DII score and DM is controversial. Some studies suggest a positive relationship between the DII score and DM (13–15), whereas other studies indicate no positive association (16, 17) or a negative association (18). To date, only one narrative review by Phillips et al. has assessed the relationships between DII and DM (19), but no systematic review and meta-analysis has been reported. A systematic review and meta-analyses using published study data may be of great value with respect to clarifying the associations between DII and DM and may provide a theoretical basis for dietary management in DM patients.

## Materials and Methods

The current systematic review and meta-analysis was conducted in accordance with the Preferred Reporting Items for Systematic Reviews and Meta-Analyses guidelines (20).

### Search Strategy

Relevant studies published until February 1, 2021 pertaining to DII and DM were searched for in the online databases PubMed, Embase, and Web of Science. In the current study, articles were identified by utilizing the following keywords: “DII” OR “dietary inflammatory index” OR “diet” OR “dietary” in combination with “diabetes mellitus” OR “diabetes.” The literature search had no limitations on country or race. Additionally, all reference lists in the articles identified were manually checked in an effort to locate any missed relevant papers. The full texts of all the articles located were read to determine whether they met the inclusion criteria. All the above processes were conducted independently by two authors. Any discrepancies were resolved by reaching a consensus *via* discussion.

### Inclusion and Exclusion Criteria

Publications were considered eligible if they met the following criteria: (i) studies conducted using adult subjects; (ii) studies that reported odds ratios (ORs), hazard ratios (HRs), or risk ratios (RRs) with 95% confidence intervals (CIs) for the highest to the lowest DII scores; (iii) studies that reported definitions of outcomes; and (iv) studies with either a cohort, case-control, or cross-sectional design. Studies were excluded if they were (i) studies on other diseases or dietary patterns; (ii) studies without complete data; (iii) dietary pattern (Mediterranean diet); (iv) review papers; or (v) so-called “gray” literature (dissertations, evaluations, working papers, book chapters, conference abstracts, and interviews).

### Data Extraction

Data were extracted from the reports and summarized by one reviewer, and then another reviewer independently checked the accuracy of that information. Data extracted from original articles included (i) study characteristics (the first author’s name, year of publication, country, study design), (ii) participant information (sex, age), (iii) follow-up duration, (iv) sample size, (v) number of cases, (vi) DII measure and dietary assessment tool, (vii) risk estimates with CIs, and (viii) adjustments. In cases of missing data, the first author was contacted in an effort to obtain the missing information.

### Methodological Quality Assessment

The quality of the studies included was assessed based on the Newcastle-Ottawa scale, which is a useful tool for estimating risks of bias in nonrandomized studies. The scale has three main grouping items: selection, comparability, and outcome (cohort study) or exposure (case-control study). The maximum total score is 9, and the respective maximum scores for selection, comparability, and outcome or exposure are 4, 2, and 3. A score of >5 points is considered to indicate high study quality. This step was also conducted by two authors independently, and any discrepancy was resolved by reaching a consensus *via* discussion.

### Statistical Analysis

HRs, RRs, or ORs extracted from the reports were pooled with random effect models, and then summary ORs were calculated by comparing the relationships between the two extreme categories of DII scores and DM outcomes, or using continuous DII measures when feasible. The I^2^ index was used to evaluate the presence and severity of between-study heterogeneity. I^2^ values ≤ 25%, 25%–50%, 50%–75%, and >75% respectively represented no, small, moderate, and high heterogeneity. Given the existence of moderate heterogeneity (I^2^ > 50%), subgroup analyses were conducted based on the following stratification: body mass index (BMI), age, dietary assessment tool, location, BMI adjustment, physical activity adjustment, and energy adjustment. A sensitivity analysis was performed to examine the robustness of the results by repeating the meta-analysis an additional five times, each time with one of the five studies excluded. Egger’s regression test was conducted to assess the publication bias for each outcome. All of the above statistical analyses were conducted using the STATA version 15.1 software.

## Results

### Literature Research

**Figure 1:**
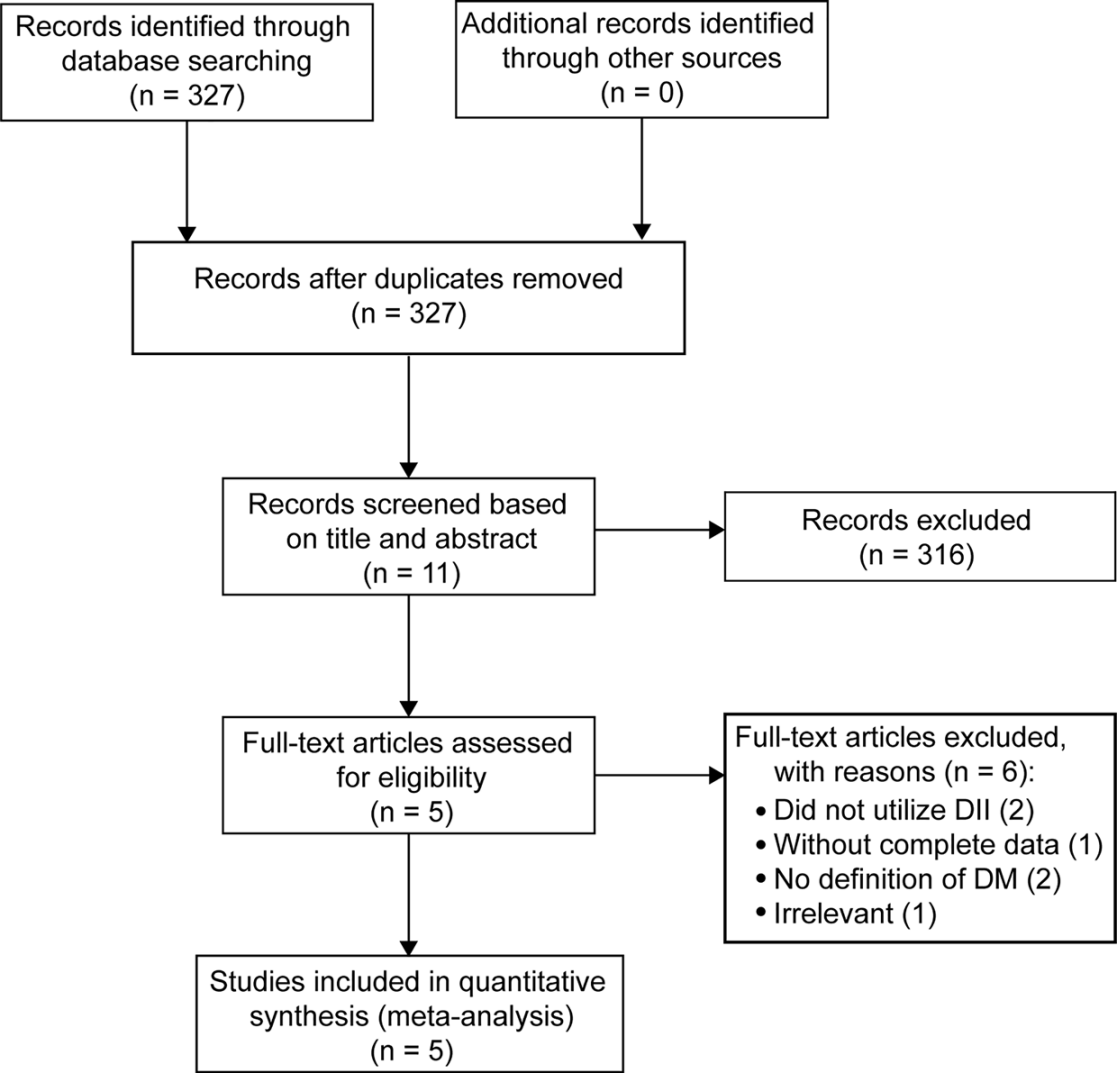
PRISMA flowchart describing the search and selection of relevant studies.

A total of 327 records were initially identified *via* database searching. After screening titles and abstracts, 316 were initially eliminated based on the inclusion and exclusion criteria, or because they were duplicates. After further assessment by reading the full texts, a further six articles were excluded because they did not utilize the DII (one study), had incomplete data (one study), did not include an accurate definition of DM (two studies), or were irrelevant (one study). Five studies ultimately met the inclusion criteria and were merged to evaluate associations between the two extreme categories of the DII score and risk of DM (13–17). The flow of the above-described procedures is shown in **Figure 1**.

### Study Characteristics

Five studies were included in the meta-analysis, involving a total of 14,987 participants and 1,366 cases from various countries, including the USA (two studies), Iran (two studies), and Mexico (one study). All five studies were published in the years from 2016 to 2020 inclusively (the DII was first introduced in 2009) (6). With regard to study design, three studies had a cross-sectional design, one was a prospective cohort study, and one had a case-control design. The sample sizes ranged from 388 to 6,016 participants, with mean ages ranging from 31.0 to 52.3 years. Follow-up duration was reported in the prospective cohort study and the case-control study, but not in any of the three cross-sectional studies. All the studies utilized either FFQs (three studies) or dietary records (two studies) as a dietary assessment tool to calculate DII scores. DII scores were used to assign study participants into a highest group (most proinflammatory diet) and a lowest group (most anti-inflammatory diet) to estimate the relationship between the DII score and the risk of DM. Adjusted ORs/RRs/HRs were reported in all five studies, and they were controlled for different types of confounding factors such as age, sex, and energy intake. Not all studies categorized DII measures into highest and lowest groups, and the study conducted by Dana et al. only reported continuous data for overall measures, not for stratified measures. In three of the five studies, there was a positive association between the most proinflammatory diet versus the most anti-inflammatory diet and the incidence of DM. Notably, however, in the remaining two studies, there was no such statistically significant association. Detailed information about the five studies in the analyses is presented in **Table 1**. According to the Newcastle-Ottawa scale, the methodological quality of all five studies was high (scores ≥ 5 points) (**Table 2**). Their findings on the association between the DII score and DM were inconsistent.

**Table 1:** Main characteristics of the studies included in the systematic review.

Author, Year	Country	Study Design	Sex	Age (y)	BMI	No. of Cases/Controls	Follow-up (y)	Outcome Assessment	Comparison	OR, RR or HR(95%CI)	Adjustments
Edger, 2018 (13)	Mexico	Cross-sectional	M/F	52.3	28.8	201/1174	–	SFFQ	Q5 *vs.* Q1 (+4.12 *vs.* -5.49)	3.02 (1.39, 6.58)	Age, sex, BMI, physical activity
Dana, 2019 (14)	USA	Cross-sectional	M/F	49.4	29.3	624/4434	–	24h-dietary record	0.79 *vs.* 0.50	1.13 (1.02, 1.24)	Age, gender, race, BMI, physical activity, smoking status, alcohol use, and socioeconomic status
Shivappa, 2019 (15)	Iran	Case-control	F	29.7	24.6	122/388	1	FFQ	Tertile3 *vs.* Tertile1	2.10 (1.02, 4.34)	BMI, age, energy, gestational age, exercise, history of diabetes, history of exposure
Mark, 2020 (16)	USA	Cohort	M	48.3	26.1	336/6016	6.5	3-day diet record	Q4 *vs.* Q1 (+1.36 *vs.* -2.60)	1.29 (0.89, 1.88)	Age, physical activity, energy intake, smoking status, alcohol use, family history of diabetes, hypertension, hypercholesterolemia
Nazanin, 2016 (17)	Iran	Cross-sectional	M/F	45	27.9	286/2975	–	FFQ	Q4 *vs.* Q1 (+1.24 *vs.* -3.43)	0.98 (0.66, 1.47)	BMI, age, smoking status, physical activity, a family history of diabetes hypertension, lipid lowering medications

RR, risk ratio; HR, hazard ratio; OR, odds ratio; DII, Dietary Inflammatory Index; FFQ, food frequency questionnaire; BMI, body mass index; M, male; F, female.

**Table 2:** Methodological quality assessment scores of the included studies.

First Author	Selection	Comparability	Outcome	Total score
Edger et al.	3	1	2	6
Dana et al.	2	2	3	7
Shivappa et al.	3	2	2	7
Mark et al.	2	2	3	7
Nazanin et al.	3	2	1	6

### Higher DII Score and Increased Risk of DM

**Figure 2:**
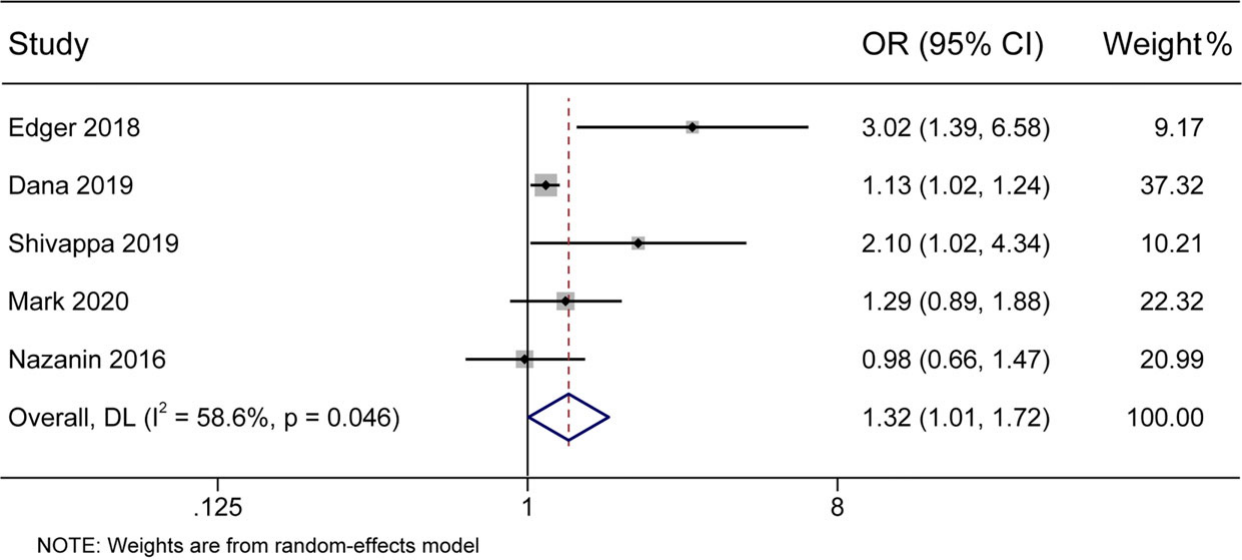
Forest plot showing the association between the DII and the risk of diabetes mellitus. Higher DII scores were associated with an increased incidence of diabetes mellitus.

In the present meta-analysis, there was a significant association between the DII score and the incidence of DM (pooled OR 1.32, 95% CI 1.01–1.72) with moderate heterogeneity (I^2^ 58.6%, *p* < 0.05) (**Figure 2**). Further subgroup analyses were conducted to identify the main possible source of the heterogeneity observed between the results of the five studies.

### BMI

A higher DII score predicted a greater risk of DM, especially in individuals with a BMI ≥ 28 (pooled OR 1.71, 95% CI 0.66–4.42, I^2^ 83.5%, *p* = 0.014) (**Table 3**). Because obesity is associated with a variety of metabolic disorder risk factors including high blood pressure, high cholesterol, and insulin resistance (IR), obese individuals are more susceptible to the effects of proinflammatory diets (21).

**Table 3:** Subgroup analyses of the association between the DII score and diabetes mellitus. Risk estimates refer to the highest *vs.* lowest DII categories.

Subgroup	Number of study	Pooled effect size (95% CI)	P (heterogeneity)	I^2^ (%)
**Total**	5	1.32 (1.01,1.72)	0.048	58.6
**BMI**				
≥28	2	1.71 (0.66, 4.42)	0.014	83.5%
<28	3	1.27 (0.89, 1.80)	0.183	41.1%
**Age**				
≥49 years	2	1.71 (0.66, 4.42)	0.4033	83.5%
<49 years	3	1.27 (0.89, 1.80)	0.048	41.1%
**Assessment tool**				
Dietary record	2	1.14 (1.04,1.25)	0.502	0%
FFQ	3	1.73 (0.84,3.56)	0.018	75%
**Location**				
America	3	1.38 (0.95, 2.01)	0.041	68.8%
Asia	2	1.35 (0.65,2.82)	0.071	69.3%
**BMI adjustment**				
adjusted	4	1.39 (0.96, 2.03)	0.025	67.8%
non-adjusted	1	1.29 (0.89, 1.88)	NA	NA
**Physical activity adjustment**				
adjusted	4	1.24 (0.95, 1.61)	0.071	57.3%
non-adjusted	1	2.10 (1.02, 4.33)	NA	NA
**Energy adjustment**				
adjusted	2	2.48 (1.46,4.22)	0.503	0
non-adjusted	3	1.13 (1.03,1.24)	0.616	0

### Dietary Assessment Tool

Stratification *via* a dietary assessment tool and age partly reduced the heterogeneity between studies, and the association differed significantly between studies that used FFQs (pooled OR 1.73, 95% CI 0.84–3.56, I^2^ 75%, *p* = 0.018) and those that used dietary records (pooled OR 1.14, 95% CI 1.04–1.25, I^2^ 0, *p* = 0.502).

### Energy Intake Adjustment

Among the variables investigated, adjustment for energy intake (pooled OR 2.48, 95% CI 1.46–4.22, I^2^ = 0, *p* = 0.503) was critical for achieving significant results as compared to studies that did not utilize such adjustment (pooled OR 1.13, 95% CI 1.03–1.24, I^2^ 0, *p* = 0.616). Because higher energy intake reflects greater food intake that could be variably distributed among proinflammatory and anti-inflammatory-associated foods, paying no attention to total energy intake may lead to bias.

### Sensitivity Analysis

**Figure 3:**
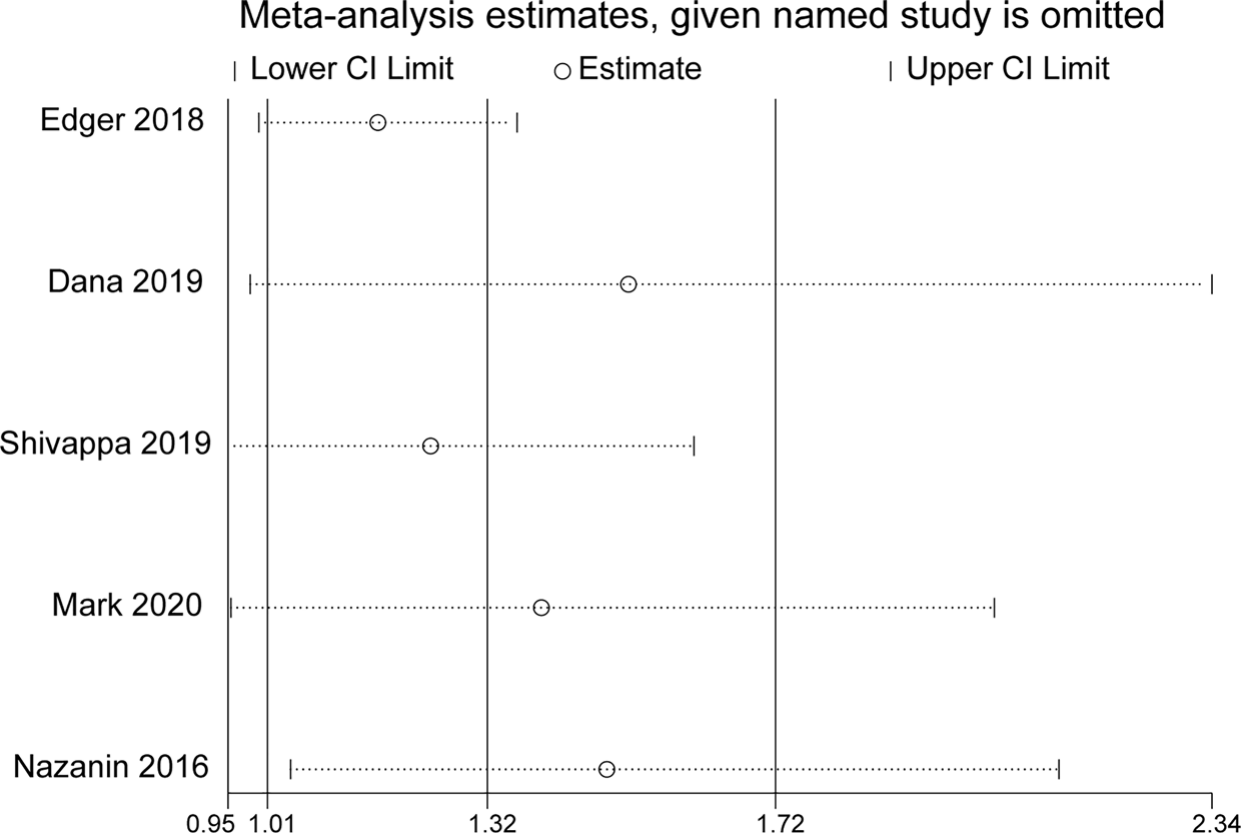
Sensitivity analysis of all included studies.

In sensitivity analysis, the overall effect estimates were not considerably altered by removing any one of the five studies (**Figure 3**).

### Publication Bias

The results of Egger’s test (t = 1.70, *p* = 0.187) indicated that there was no obvious publication bias in the five studies.

## Discussion

In the present meta-analysis, individuals with a more proinflammatory diet were at a 32% higher risk of DM. Similarly, in a review by Catherine et al., it was concluded that the DII can be used as an effective tool to evaluate the overall inflammatory nature of diet, and that it helps to identify relationships between diet, inflammation, and DM. However, that review only reported qualified synthesis, whereas the quantitative rate of the relationship remains unclear (19). On the whole, previous evidence about potential associations between DII scores and DM has been limited largely because of a lack of studies, small sample sizes, and differences in participants’ characteristics including age, sex, and race. The present systematic review and meta-analysis incorporated results from five published studies and, thus, identified stronger associations.

Diet is widely believed to be a main contributor to chronic inflammation (5). A Western diet is generally rich in proinflammatory foods such as sugar, red meat, dairy products, refined carbohydrates, and fried foods, and this may increase the serum levels of inflammatory markers (22) and the risk of DM in both males (23) and females (24). In contrast, a Mediterranean diet is generally rich in vegetables, olive oil, and nuts, and low in red meat, with poultry and fish replacing beef and lamb. Several epidemiological studies have assessed the protective effects of a Mediterranean diet with respect to several diseases associated with chronic low-grade inflammation such as cancer, diabetes, obesity, metabolic syndrome, and atherosclerosis (25). Therefore, we investigated and summarized the current evidence on associations between several normal dietary patterns and inflammation, as derived from epidemiological studies (26–31) (**Table 4**).

**Table 4:** Effects of normal dietary patterns on inflammation.

Dietary type	Dietary pattern component	Effect
Mediterranean diet	Rich in vegetables, olive oil and nuts and low in red meat with poultry and fish replacing beef and lamb.	Pro-inflammatory
Nordic diet	Based on fruits, vegetables, potatoes, fresh herbs, plants, mushrooms, nuts, whole grains, meats from	Pro-inflammatory
Tibetan diet	Focusing on high protein and vitamin rich food	Pro-inflammatory
DASH diet	Rich in fruit, vegetables, lower fat dairy, lean meat, and whole grains	Pro-inflammatory
Atkins diet	High fat, low carbohydrate diet	Pro-inflammatory
Western diet	Rich in sugar, red meat, dairy products, refined carbohydrate and fried foods, low in fruits, vegetables, nuts, fish, legumes and whole grains	Anti-inflammatory
Ornish diet	High carbohydrate, low fat diet	Anti-inflammatory
Zone diet	High protein diet	Not clear

DASH, dietary approaches to stop hypertension.

The correlation between diet and DM risk can be partly explained by chronic inflammation that may lead to IR and β cell dysfunction (32). The medium function of inflammation may involve multiple mechanisms, as described below. Activation of c-Jun N-terminal kinase by the excessive production of inflammatory factors induces phosphorylation of insulin receptor substrate-1 (IRS-1) at Ser307 leading to its inactivation, thus suppressing insulin receptor signaling (33, 34). Inflammatory factors also reduce insulin-dependent glucose transport and peripheral glucose utilization respectively by inducing the suppressor of cytokine signaling-3 and inhibiting lipoprotein lipase activity (35). Oxidative stress damage induced by inflammation may cause β cell apoptosis and deficiency of insulin secretion. In addition, sustained activation of mTORC1/S6K1 under conditions of nutrient overload (glucose, amino acids—especially branched-chain amino acids such as leucine) *via* AMP-activated protein kinase generates a feedback loop that suppresses IRS-1 mRNA and protein levels and IRS-1 activity. mTORC1/S6K1 plays a dual role in β cells, regulating β cell size and inhibiting insulin/insulin-like growth factor 1 signal transduction, which may lead to increased apoptosis (36, 37).

The original DII was created as a useful tool for categorizing the diets of individuals on a continuum from maximally anti-inflammatory to maximally proinflammatory, and it can be used in epidemiologic studies to estimate the potential burden of DM related to diet. All dietary indices prior to the DII such as the Healthy Eating Index, Alternative Healthy Eating Index, Dietary Approaches to Stop Hypertension, and Mediterranean Dietary Index were not specifically designed to evaluate the overall inflammatory potential of diet (38–42). In contrast, the DII was developed to reflect all evidence from a wide range of populations as well as laboratory animal and cell culture experiments.

The results of subgroup analyses indicated that individuals with a higher BMI are more susceptible to the effects of inflammatory stimuli. The strong associations between obesity, inflammation, and DM can be partly explained by the recruitment of macrophages into adipose tissue. Excessive inflammatory factors produced by macrophages and adipocytes may lead to the activation of c-Jun N-terminal kinase and subsequently inhibit phosphorylation of IRS-1 serine residues that induce IR  (34). Studies that used FFQs identified stronger associations than those that used dietary records. This may be because DII scores can be affected by dietary patterns and dietary assessment tools. Although completing an FFQ requires more time than completing a dietary record, it is more accurate, enabling investigators to estimate relatively long-range dietary intake, focus on specific foods or nutrients consumed by a given population, and understand relationships between diet and health outcomes (43, 44).

The current meta-analysis had several limitations. Only five studies were eligible for the analysis of DM risk. Because the DII was first created in 2009, to date, few studies have used it, particularly studies investigating associations between DII scores and DM. Different DII score categorical cutoff points (such as quintile and quartile) rendered comparisons less straightforward; thus, we could only obtain a quantitative rate of the association between the DII and DM but not a specific range. As a part of individual investigations and the basis of DII scores, the availability of FFQ data is offset by well-documented limitations such as recall bias, limitations associated with assessing specific cultural food items, and lack of validation in different research settings. Lastly, substantial heterogeneity was observed in the current meta-analysis. One possible explanation for this is the differences in energy intake adjustment used in the studies included. Another may relate to the different demographic characteristics and designs of the studies. Despite the above-described limitations, the DII calculation method used was the same in each study, thus increasing comparability. The present meta-analysis also provides greater statistical power and a more robust pooled estimate of associations by pooling results from all of the included studies.

## Conclusion

Diets resulting in high DII scores are associated with an increased risk of DM, particularly in obese individuals. However, the results of the current meta-analysis were based on a small number of studies, and further high-quality prospective studies with larger sample sizes conducted in different populations and countries are required to elucidate this association.

## Data Availability Statement

The original contributions presented in the study are included in the article/supplementary material. Further inquiries can be directed to the corresponding authors.

## Author Contributions

YX and C-LG designed this study. Q-QT and X-YD extracted and confirmed the data. Q-QT performed the statistical analysis. Q-QT was responsible for manuscript preparation. YX and C-LG commented on the manuscript. All authors contributed to the article and approved the submitted version.

## Funding

This work was supported by the Natural Science Foundation of China (NO.81900764, NO.81970676), Sichuan Science and Technology Program (NO.2019YFS0537, NO.2020YFS0456), and Luzhou-Southwest Medical University cooperation project (NO.2020LZXNYDJ32).

## Conflict of Interest

The authors declare that the research was conducted in the absence of any commercial or financial relationships that could be construed as a potential conflict of interest.

## Publisher’s Note

All claims expressed in this article are solely those of the authors and do not necessarily represent those of their affiliated organizations, or those of the publisher, the editors and the reviewers. Any product that may be evaluated in this article, or claim that may be made by its manufacturer, is not guaranteed or endorsed by the publisher.

## Abbreviation

DII, dietary inflammatory index; DM, diabetes mellitus; BMI, body mass index; IR, insulin resistance; NOS, Newcastle-Ottawa scale; FFQ, food frequency questionnaire; WPD, western pattern diet; IRS-1, insulin receptor substrate-1; SOCS-3, suppressor of cytokine signaling-3; LPL, lipoprotein lipase; JNK, c-Jun N-terminal kinase; WOSCOPS, West of Scotland Coronary Prevention Study; HEI, Healthy Eating Index; AHEI, Alternative Healthy Eating Index; DASH, Dietary Approaches to Stop Hypertension; MDI, Mediterranean Dietary Index; LPL, low-density lipoprotein.
